# A Study of Distributed Earth Observation Satellites Mission Scheduling Method Based on Game-Negotiation Mechanism

**DOI:** 10.3390/s21196660

**Published:** 2021-10-07

**Authors:** Lihao Liu, Zhenghong Dong, Haoxiang Su, Dingzhan Yu

**Affiliations:** 1 Graduate School, Space Engineering University, Beijing 101416, China; liulihao070204@163.com (L.L.); lelouchzero1221@163.com (H.S.); ardean315@yahoo.com (D.Y.); 2 School of Space Information, Space Engineering University, Beijing 101416, China

**Keywords:** EO satellite, distributed satellite system, distributed mission scheduling

## Abstract

While monolithic giant earth observation satellites still have obvious advantages in regularity and accuracy, distributed satellite systems are providing increased flexibility, enhanced robustness, and improved responsiveness to structural and environmental changes. Due to increased system size and more complex applications, traditional centralized methods have difficulty in integrated management and rapid response needs of distributed systems. Aiming to efficient missions scheduling in distributed earth observation satellite systems, this paper addresses the problem through a networked game model based on a game-negotiation mechanism. In this model, each satellite is viewed as a “rational” player who continuously updates its own “action” through cooperation with neighbors until a Nash Equilibria is reached. To handle static and dynamic scheduling problems while cooperating with a distributed mission scheduling algorithm, we present an adaptive particle swarm optimization algorithm and adaptive tabu-search algorithm, respectively. Experimental results show that the proposed method can flexibly handle situations of different scales in static scheduling, and the performance of the algorithm will not decrease significantly as the problem scale increases; dynamic scheduling can be well accomplished with high observation payoff while maintaining the stability of the initial plan, which demonstrates the advantages of the proposed methods.

## 1. Introduction

Driven by the need of more accurate, reliable, and frequent data to study earth science related issues, space engineering industry [1,2,3,4] is facing an architectural change of paradigm. Distributed Satellite Systems (DSS) encompassing several interacting spacecraft are leading the way in applications where monolithic satellites have become obsolete in terms of risk, cost, or even performance [5]. One typical instance is distributed earth observation satellites (DEOS), which involves fleet of satellites to detect the Earth’s surface and lower atmosphere to obtain information [6]. Though with more satisfied revisit times, coverage of larger areas and higher resolution, DEOS constellations are facing new challenges, the core of which is how to coordinate the mission plans for numbers of satellites that are generally heterogeneous [7].

During the past decades, researchers have carried out extensive research on centralized methodologies. Satellites mission planning are usually designed to be executed on the ground with the premise that all the observation requests and resource information are updated in a centralized way. Researchers use methods such as ant colony algorithm [8,9,10], simulated annealing algorithm [11,12], genetic algorithm [13,14,15], tabu search algorithm [16,17,18], etc. to handle the scheduling problem. Although optimal solutions can be obtained under certain circumstances, it is not practical to apply the methods above directly in distributed satellite mission planning due to the following drawbacks. Firstly, computation and memory cost could be extremely expensive as the number of satellites and observation requests increase. Time consumption in one planning period is so high that it cannot satisfy the need for rapid response. Secondly, there is always a time delay between satellites and ground stations; when emergencies (resource malfunctioning, observation failure, etc.) occur onboard, it will take time for mission planning center (MPC) to be informed, which leads to missed observation opportunities. Moreover, these methods always have difficulties in handling situations where observation requests are generated online. Whenever changes occur, the planning process should be resumed completely, which may create conflict with the executed missions in progress and lead to waste of time and resources. Finally, this kind of architecture does not fit the trend of decentralized way of system management and control, and malfunction in the central point may lead to the whole system’s failure. Consequently, new methods using a self-organized mechanism to make DSS work in a more resilient, flexible, and adaptable way are in urgent need, by which a system-level optimal can be reached when agents in the system cooperate with each other based on local and exchange information.

To address the issue above, modeling DSS as multi-agent systems (MAS) is a common trend in many experimental and academic works. The European Space Agency [19] (ESA) has implemented multi-agent system (MAS) technologies in Earth Observation (EO) constellations. Unlike traditional heuristic methods that are well-suited and designed for a fixed number of missions and satellites, multi-agent methods allow for an adaptability and scalability that is not comparable [20]. Other similar work [21,22,23] shows how MAS-related applications can improve system performance and provide negotiation-based self-organizing mechanisms as means to enhance the autonomy of a system. We can find that, after implementing the MAS framework, the definitions of coordination rules or mechanisms can directly determine the quality of DSS. Luckily, game theory [24,25,26], which focuses on cooperation and conflict between rational players, can be perfectly utilized in this kind of problem, and we can see considerable applications of game theory, including unmanned aerial vehicle (UAV), sensor web, electricity management, job scheduling, etc. For example, study [27] introduces game theory based flight control algorithms run by each autonomous UAV to satisfy the need of speedy and dynamic adjustments to swarm operations. Study [28] addresses the cooperation problem in underwater acoustic sensor network (UASN) using game theory-based clustering scheme to balance network energy consumption. Study [29] presents a real-time implementation of a multi-agent-based game theory model for microgrid market operations to monitor, control, and perform the reverse auction process of distributed energy resources (DER). In addition, as more complex systems recently emerged in the real world, networked game theory also gained prominence and showed great potential for application in socio-ecological systems [30], networked systems [31], project management [32], etc., but surprisingly, relatively little study has focused on implementing game theory in DEOS.

Moreover, dynamic scheduling which focuses on quick response to emergency observation requests, is also a practical issue for EOS applications. Perberton et al. [33] listed four key dynamic elements that can make an impact on change of satellite scheduling plan, namely, mission observation opportunity changes, resource changes, new mission insertion, and scheduling parameter changes, but they did not propose any related models or algorithms. Other related studies seem to reveal a major concern regarding this problem, which is how to consider the adjustability brought by those dynamic elements to the initial plan. Some researchers [34,35] have implemented a complete reprogramming algorithm to solve this problem, but the new plan generated is greatly different from the original one, which may create conflict with the executed missions in progress and lead to waste of time and resources. Cui [16] proposed a multi-satellite dynamic mission scheduling model and used a dynamic scheduling algorithm based on mission priority. When a new mission arrives, this approach will respectively conduct insert, reallocation, and replacement operations to reschedule the plan according to mission priority. Other similar works [36,37,38] propose some mechanisms or strategies to solve the dynamic scheduling problem; overall, these methods reduce the complexity of the dynamic adjustment and maintain the stability of the initial scheduling plan, but the efficiency and scalability can be further improved.

The overall goal of this paper is to design a distributed mission scheduling architecture that can meet the mission-level scheduling needs of heterogeneous multi-satellite earth observation system in both static and dynamic environments. Specifically, we see each satellite as a rational player and convert the satellite mission scheduling into a cooperative game. Aiming at finding a near global optimal solution in a decentralized way, this paper presents a distributed mission scheduling model, which guarantees convergence to a Nash equilibrium. Moreover, we propose a distributed mission scheduling algorithm along with two improved optimization algorithms for satellite mission scheduling in both static and dynamic environments. Finally, case studies are presented to demonstrate the effectiveness of the proposed methods.

## 2. DEOS Mission Scheduling Model

### 2.1. Problem Description

The purpose of DEOS mission scheduling is to allocate satellite resources to observation requests to maximize observing revenue while minimizing the resource consumption. Satellite earth observation activities are affected by satellite orbits, payload parameters, platform maneuvering, and other factors. A satellite’s access to the area has periodic characteristics, and only a limited area can be observed at the same time. When the number of satellites and the demands for user observations increase, multi-satellite mission scheduling will turn into a complex combination optimization problem. In order to utilize satellite resources adequately and efficiently, a satellite mission scheduling system must take both resources and user needs into cautious consideration beforehand and output a reasonable scheduling plan without conflicts. For future reference, main notations are defined in Table 1.

Considering the actual DEOS system, this paper makes the following reasonable simplifications and assumptions for the mission scheduling process:

(1) The observation task involved in this paper refers to the observation of point targets on the ground by satellites using different types of payloads, and a target only needs to be observed once.
(2) A single satellite carries only one payload. This paper considers two types of payloads, visible light and synthetic aperture radar (SAR). The satellite only executes one mission at one time. Once the mission starts, no interruption is considered.
(3) Each satellite has computing and processing capabilities, and there is a real-time communication link between the satellites, which can meet the needs of mutual communication and information transmission at any time.
(4) The situation of satellite orbit maneuver is not considered.

### 2.2. Scheduling Problem Modeling

#### 2.2.1. Model Constrains

On the basis of the above symbolic parameters, the constraints considered in this paper include:

(1) Visible window constraints. The satellite payload must be visible to target, and the window duration must not be less than the mission observation time.

(1) $$\begin{array}{c} \forall i\in \{1,\ldots ,n\},j\in \{1,\ldots ,m\},st_{i}{}^{j}\in St,\exists w^{k}\in W\\ sbt_{i}{}^{j}\geq wbt^{k},set_{i}{}^{j}\leq wet^{k},Dur_{i}{}^{j}\leq wdt^{k} \end{array}$$

(2) Slew angle constraints. During task switch, the slew angle cannot exceed the maximum slew angle of satellite.

(2) $$\begin{array}{c} \forall i\in \{1,\ldots ,n\},j\in \{1,\ldots ,m\}\\ sa_{i}{}^{j}\leq sa_{i} \end{array}$$

(3) Task preparation time constraints. The interval between two tasks (the time interval from the end of the previous task to the beginning of the next task) must not be less than the payload preparation time.

(3) $$\begin{array}{c} \forall i\in \{1,\ldots ,n\},j\in \{1,\ldots ,m\},st_{i}{}^{j},st_{i}{}^{j+1}\in St\\ sbt_{i}{}^{j+1}-set_{i}{}^{j}\geq Pre_{i}{}^{j+1}\\ Pre_{i}{}^{j+1}=(sa_{i}{}^{j+1}-sa_{i}{}^{j})/Sas_{i} \end{array}$$

(4) Energy constraints. The accumulation of energy consumed by a single satellite to execute tasks and switch tasks cannot exceed the upper limit of the energy storage of the satellite.

(4) $$\begin{array}{c} \forall i\in \{1,\ldots ,n\},j\in \{1,\ldots ,m\}\\ \sum_{j=1}^{num_{i}}P_{i}{}^{j}\leq pow_{i}\\ P_{i}{}^{j}=Dur_{i}{}^{j}\times Pt_{i}+Pre_{i}{}^{j}\times Ps_{i} \end{array}$$

(5) Storage capacity constraints. The accumulation of storage consumed by a single satellite to execute tasks cannot exceed the upper limit of the storage capacity of satellite.

(5) $$\begin{array}{c} \forall i\in \{1,\ldots ,n\},j\in \{1,\ldots ,m\}\\ \sum_{j=1}^{num_{i}}D_{i}{}^{j}\leq sto_{i} \end{array}$$

(6) Lighting constraints. This paper mainly considers two types of payloads: visible light camera and synthetic aperture radar. SAR can be observed in the full orbital period, and visible light camera can only observe within a certain sun elevation angle. The sun elevation angle needs to meet the following formula.

(6) $$\begin{array}{c} \forall l\in \{1,\ldots ,k\},i\in \{1,\ldots ,n\}\\ Sun(l)\geq SunAng_{i} \end{array}$$

(7) Payload type constraints. The target must be observed by the payload of the required type.

(7) $$\begin{array}{c} \forall i\in \{1,\ldots ,n\},j\in \{1,\ldots ,m\},st_{i}{}^{j}\in St\\ Payl_{i}=Req_{j} \end{array}$$

(8) Resolution constraints. The resolution of satellite observing the target should not be lower than the users’ requirement.

(8) $$\begin{array}{c} \forall i\in \{1,\ldots ,n\},j\in \{1,\ldots ,m\},st_{i}{}^{j}\in St\\ sr_{i}\leq tr_{j} \end{array}$$

#### 2.2.2. Scheduling Solution

**Figure 1:**
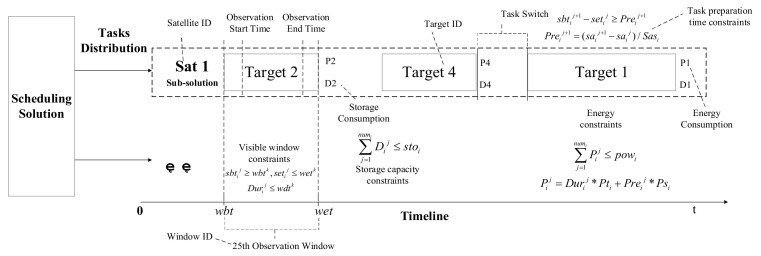
Scheduling solution.

The purpose of scheduling is to find an optimal execution plan for satellites that can maximize the payoff of observing targets. From the scheduling plan, the tasks of a satellite can be arranged in a timeline, and each satellite will know exactly the execution and termination time of observation, as shown in Figure 1. It is noteworthy that all the scheduling plan needs to satisfy the model constraints in Section 2.2.1 to make it executable.

During a scheduling period, observation opportunities are strictly limited by observation windows. We naturally number the observation windows and use metaheuristic algorithms to find optimal combinations so as to deconflict and output an executable scheduling plan. In this paper, we define a 6-tuple to record an observation chance to one target of a certain satellite, namely, [Window ID, Target ID, Satellite ID, Observation Start Time, Observation End Time, Observation Slew Angle]. The scheduling solution consists of numbers of (the length of solution is the number of targets) these tuples, and detailed execution information of the scheduling plan can be indexed from the observation windows set.

### 2.3. Optimization Objective of Satellite Mission Scheduling

Satellite mission scheduling is a combined optimization problem with multiple constraints and objectives. Through scheduling, we want to obtain a solution that not only prioritizes observing high-value targets (high priority) and a high task completion rate but also ensures less energy consumption among satellites. Based on this consideration, we set following sub-goals and use a linear weighted sum method to set the optimization objective for solving this model:

(1) Sub-objective 1: Maximize the priority of the target observations.

(9) $$y1=a\times Max(\sum_{i=1}^{n}\sum_{j=1}^{num_{i}}X_{i}^{j}\times prio^{j})/\sum_{j=1}^{m}prio^{j}$$

(2) Sub-objective 2: Maximize the number of target observations.

(10) $$y2=b\times Max\sum_{i=1}^{n}\sum_{j=1}^{num_{i}}X_{i}^{j}/m$$

(3) Sub-objective 3: Balance resource usage among satellites.

(11) $$y3=c\times Min\sum_{i=1}^{n}P_{i}y3=c\cdot Min\sum_{i=1}^{n}P_{i}$$

On the basis of the above three sub-goals, the total optimization goal of this paper is obtained by a linear weighted sum method.

(12) y=α×y1+β×y2+γ×y3

Due to different measurements of priority, task execution number, and energy consumption, it cannot be directly evaluated at the same time. This paper introduces the dimensional parameters *a*, *b*, and *c* to unify each index, and the values are *a* = 1/4, *b* = 1, and *c* = 1/5, in order to ensure that the benefit of observing a target must be higher than the loss of energy consumption. In equation (12), *α*, *β*, and *γ* are weight indicators, and the sum of them is 1. The value of the weight determines the importance of the sub-objective in the overall optimization goal. This paper takes the priority of the task as the key factor, and we set α = 0.7, *β* = 0.2, and *γ =* 0.1.

## 3. Distributed Mission Scheduling Model

### 3.1. Game Model of DEOS

To achieve efficient system coordination among multiple satellites, this paper sees the DEOS as a multi-agent system and converts multi-satellite mission planning into a game problem *G =* (*N*, *{A_i_}*, *{U_i_}*), where *N = S* is the set of players (agents) in a game. Every satellite *s_i_*∈*S* in this game is seen as a ‘rational player’ (agent), whose rationality is embodied in that it will continuously adjust its own action strategy *a_i_* in response to the action strategy adopted by opponents, so as to maximize its own payoff *U_i_*. Action *a_i_* is the scheduling plan that is only executed by the *i-th* satellite, and it is a subset of the overall scheduling solution defined in Section 2.2.2. As shown in Figure 1, the sub-solution for Sat1 represents the current action adopted by the 1st satellite in that scenario. In addition, *A* = ∏*_i__∈__N_ A_i_* represents the set of joint actions taken by the entire system in one round of the game. For simplicity of expression, let *a_−i_ =* (*a_1_*,*…*, *a_i−1_*, *a_i+1_*,*…*, *a_n_*) be the action profile other than *s_i_*. To terminate iterations of gaming, we introduce Nash Equilibrium to our model.

**Definition** **1.** *(Nash Equilibrium* [39]*) For a game problem G =* *(N, {A_i_}, {U_i_}), for any player i∈N, we call a∗ a Nash equilibrium, when it satisfies:*

(13) $$U_{i}(a_{i}^{*},a_{-i}^{*})=\max_{a_{i}^{}\in A_{i}}U_{i}(a_{i}^{},a_{-i}^{*})$$

In iterations of reaching Nash equilibrium of a multi-satellite game, each satellite modifies its own action without directly interfering with other satellites. Due to the optimization objective setting in Section 2.3, each satellite will try to ‘capture’ as many unscheduled targets as possible to gain a higher individual payoff and meanwhile pay close attention to targets that have been scheduled by others to avoid repeated observation. The gaming process will continue to run until the system reaches a balance when all individuals are unable to obtain higher payoffs by changing their own actions. It is noteworthy that after each round of the game, each satellite must inform other satellites of its action to maintain the consistency of cognition. In Section 2.1, we assume that each satellite with the same payload can interact in real time through inter-satellite links, which provides a basis for the future optimization based on Nash equilibrium.

### 3.2. DMSA Based on Nash Equilibrium

For the generalization of the problem, in distributed mission scheduling algorithm (DMSA, as shown in Algorithm 1), we set *n* finite memory tables of length *L* that record the historical actions adopted by each player *s_i_* and denote them by *Mem_i_^t^* = (*mem_i_^1^; mem_i_^2^; …*, *mem_i_^L^*) and (*i* = 1,2,…,*n*), respectively, whose initialization is defined to be empty. When optimization begins, every player will be randomly allocated several targets and generate its own action space *A_i_*. In each round of gaming, all the players exchange information, and each player selects its action; *a_i_∈A_i_* is by the adaptive particle swarm optimization algorithm (APSOA) in static scheduling or by the adaptive tabu-search algorithm (ATSA) in dynamic scheduling, both of which will execute constrains check and deconflict process and thus calculate current payoff *U_i_*(*t*) in (12). As Equations defined in (9)–(11) are the overall optimization sub-objectives of scheduling, when calculating current payoff *U_i_*(*t*) of each satellite in (12), the variable *n* is set as (1) and the best response *BR_i_^t^* as

(14) $$BR_{i}{}^{t}=\arg\max_{a_{i}^{t}\in A_{i}}U_{i}(a_{i}^{t},a_{-i}^{*})$$

Then, each individual will compare current *BR_i_^t^* with last *BR_i_^t−1^* (or *a_i_^t−1^*) recorded in the memory table and follow the greedy strategy to select the one with higher payoff as current action *a_i_^t^*. After recording *a_i_^t^* into the bottom of memory table as newest *mem_i_*, the DMSA will check whether the number of elements in the current memory table is greater than length *L*, and if so, *s_i_* will delete the oldest element (the *mem_i_^1^*) to obtain an updated fixed-length *Mem_i_^t^* and then reallocate those allocated but unscheduled targets to neighbors (neighboring satellites refer to satellites with the same payload, and we assume they can send information to each other directly at any time). The gaming will continue to process until recorded actions of all players’ memory tables stay consistent for *L* rounds, which means all satellites are unable to obtain higher returns through their own plan adjustments.
**Algorithm 1** DMSA**Input**: Satellites Set *S*, Targets Set *T*, memory length *L*, Observation Windows Set *W*. **Output**: Overall Scheduling Plan **Procedure**: 1.**for** each round of Game, g = 1,2,3…, **do** 2.    **for** each satellite *s_i_*∈*S*, simultaneously **do** 3.        Receive information from neighbors; 4.        Calculate and select *U_i_*, *BR_i_^t^* by APSOA/ATSA; 5.        Find received targets that haven’t been scheduled; 6.        Send unscheduled targets to neighbors; 7.        Update *Mem^i^_t_* of each *s_i_*; 8.    **end for** 9.**end for**

### 3.3. APSOA for Single Satellite Scheduling

#### 3.3.1. Solution Coding Scheme

**Figure 2:**
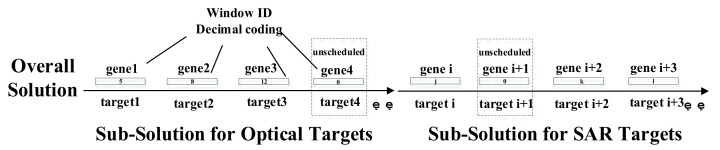
Solution coding scheme.

Coding scheme is directly related to the design of model and efficiency of algorithm. In the data preprocessing stage, observation windows defined in Section 2.2.2 are calculated and numbered, and each window can be uniquely indexed by a window ID. As observation can only happen during the window time period, an optimal scheduling solution is actually a combination of observation windows that not only satisfy the model constrains but maximize the observation payoff. In this paper, the solution coding is organized in a chromosome manner with gene fragments on it (the number of gene fragments is equal to the total number of targets). In each gene, there is a non-negative decimal number which corresponds to the observation window ID (0 indicates that the current target has not been scheduled for observation). For satellites with the same payload type, they share the same sub-solution coding (the length of sub-solution is equal to the number of targets with the required observation type), the final overall solution will be the accumulation of sub-solutions with same payload type, and connections of sub-solutions with different payload types, as shown in Figure 2.

#### 3.3.2. APSOA Procedure

In static scheduling when all observation requests are proposed beforehand, the optimization results in each round of the game will have an impact on the overall scheduling plan of the system. In order to improve the solving efficiency and final payoff of the distributed optimization algorithm, each satellite needs to output a satisfactory sub-plan in a relatively short time. This paper designs an APSOA, whose inertia weight parameter *λ* will be adaptively adjusted as the iterations evolve, so that the solving speed will gradually increase, and convergence will occur as soon as possible.

(15) $$\lambda =\lambda_{\max}-\frac{(\lambda_{\max}-\lambda_{\min})\cdot t}{T_{\max}}$$

where *T_max_* is the maximum iteration number, *t* is the current iteration number, *λ_min_* is the minimum inertia weight parameter, and *λ_max_* is the maximum inertia weight parameter. The pseudo code of the APSOA is shown in Appendix A.

#### 3.3.3. Static Scheduling Evaluation

The evaluation methods for static scheduling mainly include three indicators: the first one is the overall payoff of the scheduling plan (*Payoff*), and the second one is the mission completion rate (*CR*). These two indicators mainly reflect the profit of the satellite mission execution plan. The third one is the running time (*RT*), which mainly reflects the solving speed of the optimization algorithm.

### 3.4. ATSA for Dynamic Scheduling

Dynamic scheduling occurs when emergency missions arrive or change of initial plan is required. This section presents an ATSA to solve this problem. Before introducing the algorithm, we firstly analyze what will happen to the solution when an emergency comes up.

#### 3.4.1. Impact of Emergency on Initial Solution

##### New Mission Arrival

**Figure 3:**
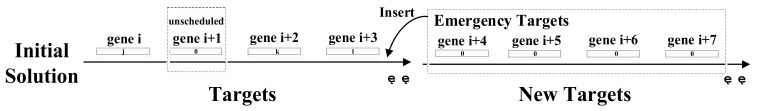
Emergency targets inserted into the initial solution.

Newly arrived missions are the most frequent cases of emergency conditions. When emergency happens, such as natural disaster monitoring, public health incidents, or when new targets with high priority appear, new targets are added to observation requests. It is unpractical to put newly arrived missions into a new target set and reschedule them as regular circumstances because the scheduling plan with regards to new targets might conflict with the initial plan in the same execution period. Therefore, new targets should be considered as a whole with old ones; in other words, new genes representing newly arrived targets will be numbered subsequently and inserted at the bottom of the initial solution; the solution structure update is shown in Figure 3.

##### Change of Scheduling Plan

**Figure 4:**
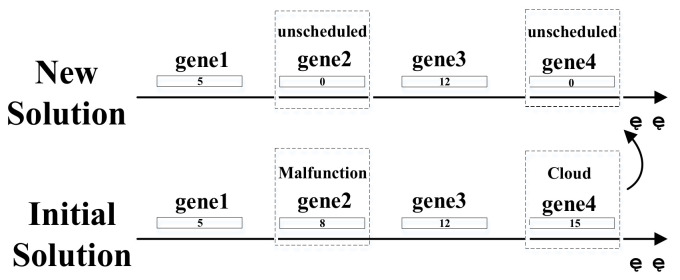
State update of scheduling plan.

Sometimes, when some targets cannot be observed as required due to weather condition or satellite resources malfunction, these targets should be rescheduled without disturbing the undergoing plan of satellites that are properly functioning. In other words, most targets can be normally executed as initially planned, while those failed missions should update their solution state (replace the window ID with 0) and reschedule, as shown in Figure 4.

#### 3.4.2. ATSA Procedure

Above all, after emergency happens, we can see that the new solution updates its structure and state based on the initial one. In dynamical scheduling, remaining satellite resources will be allocated to unscheduled tasks. Because resources between different satellites are independent of each other, the impact of the solution improvement is also limited to those satellites assigned new targets. Based on the above factors, we do not perform an entirely new round of search and optimization for every gene in ATSA but only focus on those targets that have not been scheduled. On that basis, we design a method which will call TS multiple times and adaptively update its searching strategy and solution structure (such as iteration number, neighborhood solution structure, etc.) during the optimization process. This method can be continuously trimming the search space to improve the computational efficiency as well as the quality of the solution at an acceptable cost. The pseudo code of the ATSA is shown in Appendix B.

#### 3.4.3. Dynamic Scheduling Evaluation

The goal of dynamic scheduling is to complete emergency tasks with high revenue while maintaining the integrity of initial plan. Therefore, several indicators are defined to evaluate the performance of algorithm, including the completion rate (*CR*), priority execution rate (*PR*), initial plan change rate due to newly arrived targets (*IR*), and emergency execution rate (*ER*). The evaluation function is defined as follows:

(16) $$\begin{array}{c} f=\frac{CR+PR+(1-IR)+ER}{4}\\ CR=n_{2}/n^{\prime }\\ PR=\sum_{i=1}^{n_{1}}p_{i}/\sum_{i=1}^{n^{\prime }}p_{i}\\ IR=d_{3}/n_{1}\\ ER=d_{4}/(d_{1}+d_{2}) \end{array}$$

where *n^’^* is the total number of targets, *n_1_* is the number of targets executed in the initial plan, *n_2_* is the number of targets observed after dynamic scheduling, d_1_ is the number of newly arrived targets, *d_2_* is the number of targets in the initial plan that fail to execute due to emergency, *d_3_* is the number of targets of the initial plan that change due to emergency, and *d_4_* is the total number of emergency targets that execute after dynamic scheduling.

### 3.5. Convergence Analysis for DMSA

In this section, we present theoretical analysis on convergence for DMSA. The analysis is conducted in two steps. Firstly, the existence of Nash Equilibrium should be demonstrated. It is well known that every game with a finite number of players and action profiles has at least one Nash equilibrium [39]. In a game model of DEOS, it is clear that the numbers of satellites and scheduling plans are finite, so a Nash Equilibrium must exist.

Secondly, we should prove that DMSA will stably converge to an optimal solution from any initial plan. According to the distributed optimization strategy proposed in this paper, when the inter-satellite communication is unblocked, a consensus can be reached among different satellites, and the optimization results of satellites in each round of the game are independent of each other. Therefore, after *t* rounds of gaming, the payoff *F* of the entire system is

(17) $$F=\sum_{i=1}^{N}U_{i}^{t}(a_{i})$$

From the perspective of formula (17) alone, it seems that it only needs to ensure that each individual’s payoff *U_i_^t^* is the current optimal to guarantee the overall global optimal performance. In fact, due to the “short-sighted” characteristics, each player can only obtain a local optimal solution based on the current partial information, while the accumulation of the local optimal does not necessarily represent the system-level optimal. Therefore, this article adopts the greedy rule, so that after rounds of gaming, each player will compare its *U_i_^t^* with previous action *a_i_^t−1′^*s payoff and always select one with larger payoff as new action *a_i_^t^*. Consequently, the payoff of each player must be “non-decreasing”, namely,

(18) $$\begin{array}{c} \forall i\in \{1,\ldots ,n\},\forall t\\ U_{i}^{t}(a_{i}^{t})\geq U_{i}^{t-1}(a_{i}^{t-1}) \end{array}$$

From the definition of scheduling payoff, it can be seen that the total payoff in a scenario is upper bounded. Along with the existence of Nash equilibrium, through each iteration of “non-decreasing” execution, as long as the number of iterations can be guaranteed, the distributed optimization will be stabilized. In other words, ‘stability’ means that all the individual payoffs will remain unchanged for the remaining rounds of gaming, therefore leading to a system-level balance. This is also the inspiration for setting a memory table for each satellite; the length of *L* should be set based on the overall requirements of scheduling: when *L* is small, coordination can be reached with shorter computation time, but scheduling effects rely heavily on the initial targets allocation; when *L* is large, a more satisfactory solution can be obtained with higher computation cost; when *L* is infinitely large, a Nash Equilibrium must be reached. To conclude, by setting a proper *L* in real world application, DMSA can be balanced between efficiency and effectiveness, the scheduling solution can stably converge to a local optimum, or even the global optimum.

## 4. Results and Discussion

**Figure 5:**
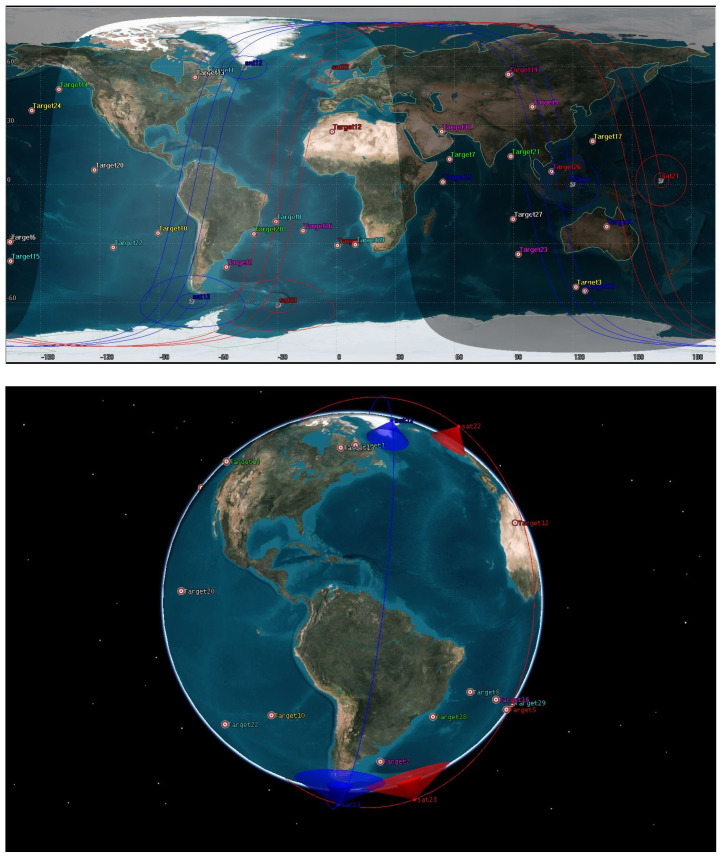
Test scenario example.

To evaluate the effectiveness of the proposed methods, this section conducts several simulation experiments. We set up test scenarios in STK software by adding different numbers and types of EOSs, payloads, and targets, as shown in Figure 5. Mission requests (including targets geographical locations, observation types, priority, resolution requirement, etc.) are generated worldwide by a random uniform distribution. During the simulation period (from 8 March 2021 16:00 to 9 March 2021 16:00, universal time coordinated), the observation satellites composed of a Walker constellation in a 600 km height sun-synchronous orbit (the inter plane true anomaly increment is 2°, the RAAN increment is 4.5°) are automatically generated. We can modify the scale of scenarios by adjusting the number of satellites in an orbital plane and orbital plane number to adjust the size of the constellation.

### 4.1. Case Study for DMSA in Static Scheduling

#### 4.1.1. Scenario Setting

The experimental cases involve several scenarios that include different numbers of satellites, different types of observations, and different distributions of observation targets. The number of mission targets ranges from 30 to 200. The target positions are randomly generated within the range of latitude −60~60°, and the observation type and priority (1~5) are randomly generated; the number of satellites ranges from 3 to 16. The type of satellites payloads in the constellation are randomly generated. The payload setting parameters are shown in Table 2.

#### 4.1.2. Algorithm Parameters Initializing

On the data preprocessing stage, the satellites and targets information in the scenario is imported into STK in batches by MATLAB, and the visible window information of payload and target is obtained through the visible analysis and calculation of STK. On this basis, the adaptive PSO algorithm is then called for task scheduling. The main parameters of the PSO algorithm are set as follows: particle population set to 50, PSO maximum iteration number set to 100, acceleration coefficients *c_1_* = *c_2_ =* 1.5, inertia weighted parameter *λ_max_ =* 0.9, *λ_min_ =* 0.4. The indication of early termination is that best fitness value of population has not been improved for 15 consecutive generations. The length of memory table *L* is set as 5.

#### 4.1.3. Typical Algorithms for Comparison

This paper selects the centralized genetic algorithm (CGA) [40], the centralized particle swarm algorithm (CPSOA) [41], and the centralized tabu search algorithm (CTSA) [16] and compares their performances in different cases with the DMSA proposed in this paper.

#### 4.1.4. Simulation Results Analysis

The simulation results of different cases are shown in Table 3. Generally speaking, with the increase in the number of satellites and the number of targets, the overall planning payoff is on the rise, but due to the increase in the complexity of the problem, the scheduling process also consumes more time.

##### Comparison and Analysis for Performances of Optimization Algorithms

**Figure 6:**
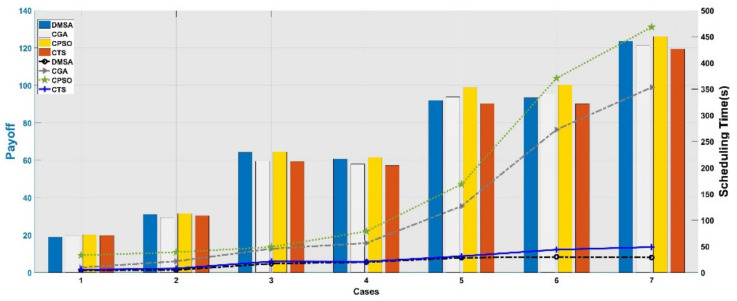
Payoff and scheduling time. Histogram shows the payoff of different algorithms’ optimization results, while the curve shows the scheduling time.

From the perspective of search strategies among mentioned algorithms, CPSOA and CGA are both global search algorithms, while CTSA focuses on searching local solution space. From Figure 6, we can see that, when the problem scale is small, there is little difference between performances of different algorithms; due to CTSA’s local search strategy, it has a considerable advantage in calculation speed over other centralized scheduling algorithms. With the increase of the problem scale, the superiority of the CPSOA’s strong global search ability is reflected. In all experimental cases, CPSOA can output a scheduling plan with the highest payoff and completion rate, while CGA and CTSA fall much more easily into a local optimum.

##### Comparison and Analysis for Scheduling Method

**Figure 7:**
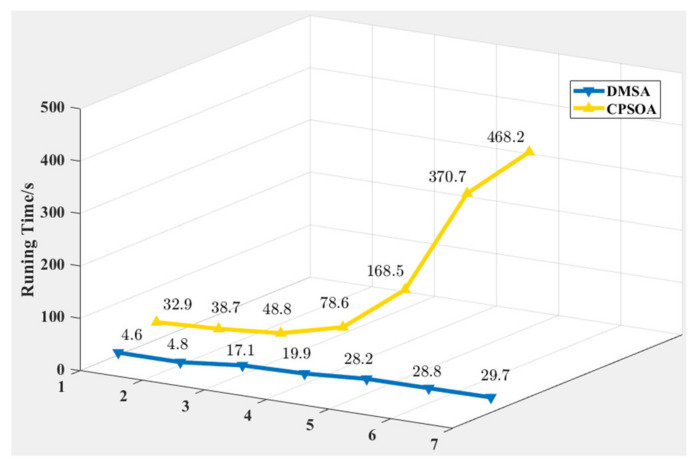
Comparison of scheduling time between DMSA and CPSOA.

From the perspective of scheduling methods, though the overall payoff will be lower than that of centralized scheduling, the distributed mission scheduling method has significant advantages in scheduling time. This is because the method of assigning targets to individuals for single-satellite scheduling is helpful in reducing the search areas of solution space, thereby improving the solving efficiency. Comparing the scheduling time between CPSOA and DMSA (as shown in Figure 7), we can see that when the scale of the problem increases, DMSA will not experience a sharp increase in scheduling time like CPSOA. Moreover, only the number of satellites increases while the number of targets remains unchanged, and the scheduling time of DMSA does not increase significantly, which fully demonstrates the advantages of the distributed system using decentralized management and control, that is, the addition or deletion of nodes will not affect the stability of the system. Individuals in the system can maintain contact and collaboration with each other through communication, while simultaneously remain relatively independent, which can greatly enhance the flexibility and robustness of the distributed system.

### 4.2. Case Study for DMSA in Dynamic Scheduling

#### 4.2.1. Scenario Setting

To verify the effectiveness of DMSA in dynamic scheduling, we set up several cases that involve emergency circumstances. In the first scenario, 30 initial point targets are randomly generated; target parameters are shown in Table 4. Moreover, another 5 high priority targets are added as emergency missions to test the dynamic scheduling algorithm, as shown in Table 5. We set a constellation of 2 orbital planes with 3 EOSs on each plane, payload types (visible light and microwave) and resolution of the EOSs are also randomly generated, as shown in Table 6.

#### 4.2.2. Algorithm Parameters Initializing

For the initialization of ATSA, the length of the tabu list is dynamically determined by the maximum number of unscheduled tasks in the current solution (larger number leads to longer length), and the neighborhood scale is set to 5. The TS global maximum iteration number is set to 150, and the local maximum iteration number is set adaptively according to times of calling different TS (later iteration leads to smaller local maximum iteration number). The global early termination indication is that the task completion rate (the proportion of scheduled task in all tasks that can be observed) reaches at least 98%.

#### 4.2.3. Simulation Results Analysis

After iterations of APSOA in static scheduling, the initial plan is output, as shown in Table 7. In the initial plan, visible light Target17 is not allocated any satellite resources because of limitation in time window (the target cannot be observed by any optical payload satellite due to lighting constraints). Then, we set an emergency circumstance where a satellite malfunction occurs: Target8, Target12, and Target21 cannot be observed as planned. Moreover, 5 emergency targets with high priority are added. After dynamic scheduling, for those initial targets with malfunction problems, microwave Target8 and Target12 are rescheduled to new satellite resources with new time windows; visible light Target30 is deleted from plan due to limited time window. For those newly arrived missions, all targets except for the visible light Target31 are directly inserted to new scheduling plan. Target31 is deleted because it cannot be observed by any satellite.

**Figure 8:**
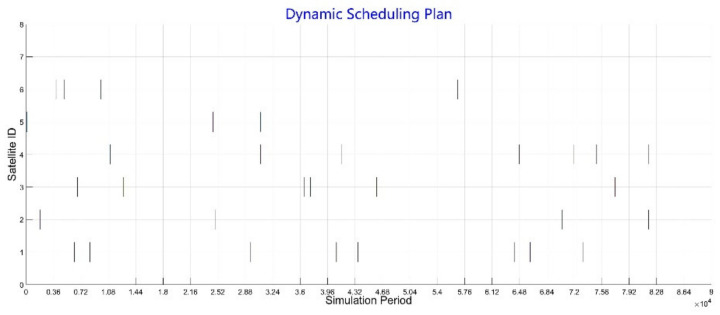
Gantt chart of dynamic scheduling.

As shown in Table 8, it can be seen that in this scenario, 29 of 30 targets are scheduled under regular circumstances. After dynamic scheduling, 91.4% of targets are observed with only 10.3% change to the initial plan, 89.3% of priorities are executed, 75% of emergency targets are allocated satellite resources, and the evaluation function value is 0.86. The number of targets executed by optical satellites (S2, S5, and S6) are 4, 3, 4, while the number of SAR satellites (S1, S3, and S4) are 8, 6, 7. It indicates that missions are allocated in a balanced way among satellites; the scheduling result is shown in Figure 8. It is noteworthy that unscheduled targets are all visible light targets, and during the whole scheduling period, Target17 and Target31 cannot be observed by any optical satellites. Moreover, because the Walker constellation is on the sun-synchronous orbit, Target30 can only be observed by S2 for once. Consequently, missed observing opportunity in the initial plan will lead to failure of observation after dynamic scheduling. Therefore, the result shows that the algorithm can efficiently make adjustments after an emergency happens and guarantee the stability of the initial scheduling plan. Targets with high priority and sufficient observation windows will be scheduled with satisfactory effect.

Several more experimental cases are conducted in this section to verify the effectiveness of the proposed method. Six scenarios with different satellites and resources are setup, and the evaluation function defined in Section 3.4.3 is performed to evaluate the scheduling results. Computational complexity is measured by the elapsed CPU time. Detailed evaluation results are shown in Table 9.

It shows that as the scale of the scenarios increases, the elapsed CPU times of both regular and dynamic scheduling exhibit an increasing trend. The average *CR* of 0.953 indicates our method can schedule most of the targets in the scenarios. It is noteworthy that *PR* is always higher than CR, and the average ER is 0.948, both of which indicate that targets with high priority are more likely to be scheduled. The *IR* is related to the proportion of emergency targets to initial targets, we can see that in the 3rd scenario where the proportion is the largest ((10 + 20)/50 × 100% = 60%), the change to initial plan after dynamic scheduling is also the largest. The running times of dynamic scheduling are much shorter than those of static scheduling, and this is because the strategy in ATSA will trim the searching space which leads to a quicker convergence.

Some may be concerned that our trimming method is reducing computational complexity at the cost of losing potential to find a global optimum, but we can see the *CR* and *PR* values are both at satisfactory level. Every time ‘old’ TS stops its iteration and updates its solutions’ structure, the ‘new’ TS searches the observation set again and tries new windows combinations of unscheduled targets. A new revenue check and de-conflict process will be calculated to see whether the scheduled targets can be substituted or deleted due to insertion of new ones, which can exploit new parts of the searching space. Moreover, the tabu list will memorize the last ‘moves’ and avoid repeated search, which helps to prevent easy convergence to a local optimal.

## 5. Conclusions and Future Work

Aiming at efficient mission scheduling for earth observation distributed satellite systems, this paper addresses the problem by learning from game theory. We firstly propose DMSA which views each satellite as a rational player that focuses on maximizing its payoff through cooperation with neighbors, and we adopt the idea of Nash Equilibrium to guarantee convergence to a near global optimal scheduling plan. To achieve static and dynamic scheduling circumstances, we propose APSOA and ATSA, respectively, and set experimental cases to evaluate their effectiveness. The simulation results show that, in static scheduling, our method can effectively overcome the shortcomings of centralized methods in large-scale mission scheduling scenarios, such as a sharp increase in scheduling time and slow convergence. The distributed mission scheduling method can flexibly deal with different scales of problem scenarios. The algorithm performance will not decrease significantly with the increase in the problem scale and can stably and efficiently obtain the near global optimal solution whose performance is slightly lower than that of the centralized scheduling. In dynamic scheduling, high priority targets will take precedence in execution, and most of the emergency targets will be dynamically scheduled in a relatively short time without major changes to the initial plan. To conclude, the proposed method yields a high scheduling revenue and low scheduling cost and provides a way of solving the DEOS scheduling problem with scalability, adaptability, efficiency, and effectiveness. It has potential to be further applied to more complex scenarios.

In future work, we will introduce new constraints and update our optimization strategy to make the scheduling model more comprehensive and robust and study the emergency demand analysis mechanism to transform user needs with precision and efficiency. Moreover, we will explore the distributed collaborative scheduling mechanism with restricted communication environments to meet the demands of more complex satellite scheduling problems in the real world.

## Figures and Tables

**Table 1:** Parameter and label definitions.

Notation	Definition	Notation	Definition
*S*	*S =* {*s_1_*, *s_2_*,*…*, *s_n_*,*…*}, where *S* represents the set of satellites, *n* represents the number of satellites.	*Payl*	*Payl =* {*Payl_1_*, *Payl_2_…Payl_i_*,*…*}, where *Payl* represents set of payloads, *i* corresponding satellite serial number.
*Dur_i_^j^*	*Dur_i_^j^* represents the imaging duration time required when the *i*-*th* satellite executes the *j*–*th* task.	*Pre_i_^j^*	*Pre_i_^j^* represents the payload preparation time required to switch from last task when the *i*-*th* satellite executes the *j*–*th* task.
*Pow*	*Pow =* {*pow_1_*, *pow_2_…pow_i_*,*…*}, where *Pow* represents the energy set of the satellite, *i* represents the corresponding satellite serial number.	*P_i_^j^*	*P_i_^j^* represents the energy consumed whenthe *i-th* satellite executes the *j–th* task, *P_i_* is the total energy consumption of the *i-th* satellite.
*Sto*	*Sto =* {*sto_1_*, *sto_2_…sto_i_*,*…*}, where *Sto* represents the storage set of the satellite, *i* represents the corresponding satellite serial number.	*D_i_^j^*	*D_i_^j^* represents the storage consumed whenthe *i-th* satellite executes the *j–th* task.
*SA*	*SA =* {*sa_1_^1^*, *sa_1_^2^…sa_i_^j^*,*…*}, where *SA* represents the slew angle set of the satellite, *sa_i_^j^* represents the slew angle when the *i*-*th* satellite executes the *j*–*th* task, *sa_i_* represents the maximum slew angle of the *i*-*th* satellite.	*Pt_i_*	*Pt_i_* represents the energy consumption per unit time of the *i-th* satellite during task execution.
*Sas_i_*	*Sas_i_* represents the slew angle change per unit time of the *i-th* satellite during task switch.	*Ps_i_*	*Ps_i_* represents the energy consumption per unit time of the *i-th* satellite during task switch.
*W*	*W =* {*w^1^*, *w^2^… w^k^*,*…*}, where *W* represents observation windows set, *k* represents the number of the windows, *w^k^* *=* [*wbt^k^*, *wet^k^*, *wdt^k^*], *w^k^* represents the *k-th* time window, *wbt^k^* represents the start time, *wet^k^* represents the end time, *wdt^k^* represents the duration.	*St*	*St =* {*st^1^*, *st^2^… st^k^*,*…*}, where *St* represents the time information set of satellites execution, *m* represents the number of tasks, *st_i_^j^ =* [*sbt_i_^j^*,*set_i_^j^*], where *st_t_^j^* represents the time information of the *i*-th satellite executing the *j–th* task, *sbt_i_^j^* represents the execution start time after schedule, *set_i_^j^* represents the end time.
*T*	*T =* {*t_1_*, *t_2_…t_m_*,*…*}, where *T* represents the set of targets, *m* represents the serial number of targets.	*Req*	*Req =* {*req_1_*, *req_2_…req_j_*,*…*}, where *Req* represents the set of observation type requests, *j* represents the corresponding target serial number.
*Num*	*Num =* {*num_1_*, *num_2_… num_i_*,*…*}, where *Num* represents the number of tasks that are executed by the satellite, *i* represents the corresponding satellite serial number.	*Prio*	*Prio =* {*prio_1_*, *prio_2_…prio_m_*,*…*}, where *Prio* represents the set of targets priority, *m* represents the serial number of targets.
*Sun*	*Sun(l)* represents the minimum sun elevation angle in *l* window period.	*maxP*	max*P* is the initial power of a satellite.
*TR*	*TR =* {*tr_1_*, *tr_2_…tr_m_*,*…*}, where *TR* represents the set of targets’ resolution requirement, *m* represents the serial number of targets.	*SR*	*SR =* {*sr_1_*, *sr_2_…sr_n_*,*…*}, where *SR* represents the set of satellites’ resolution, *n* represents the serial number of targets.
*X*	*X_i_^j^* represents the decision variables of satellites, *j* represents corresponding target serial number, *i* represents corresponding satellite serial number. Value 1 denotes that target *j* has been observed by the *i-th* satellite, value 0 denotes that the target has not been scheduled.	*SunAng*	*SunAng_i_* represents the minimum sun elevation angle of the *i-th* satellite carrying an optical payload.

**Table 2:** Payload parameters.

Parameters	Optical Payload	Parameters	SAR Payload
SensorType	SimpleConic	MinElevationAngle	15.2°
FOV	5°	MaxElevationAngle	51.9°
SlewRange	−40~40°	ForwardExclusionAngle	5.7°
Lighting	SunAng > 15°	AftExclusionAngle	8.6°

**Table 3:** Simulation results in different cases.

Case No.	Case		DMSA			CGA			CPSOA			CTSA		
	Sat	Tar	Profit	CR	RT(s)	Profit	CR	RT(s)	Profit	CR	RT(s)	Profit	CR	RT(s)
No.1	6	30	19	0.93	4.6	19.8	0.97	9.6	20.2	0.97	32.9	20	0.95	5.3
No.2	6	50	31.2	0.96	4.8	29.3	0.90	21.5	31.6	0.96	38.7	30.4	0.94	7.8
No.3	6	100	64.2	0.99	17.1	59.4	0.96	45.4	64.5	1	48.8	59.3	0.91	21.2
No.4	8	100	60.7	0.98	19.9	57.9	0.92	56.2	61.6	0.98	78.6	57.2	0.86	20.8
No.5	8	150	91.9	0.87	28.2	93.8	0.89	126.6	98.9	0.96	168.5	90.3	0.85	31.2
No.6	16	150	93.4	0.91	28.8	95.3	0.93	272.6	100.3	1	370.7	90.1	0.84	43.7
No.7	16	200	123.6	0.93	29.7	121.2	0.91	353.3	126.2	1	468.2	119.4	0.87	48.6

**Table 4:** Initial target parameters.

Target ID	Priority	Geographical Position	Observation Type	Resolution Requirement
Target1	4	(113° W, 56° N)	microwave	0.7
Target2	4	(176° W, 43° N)	microwave	0.8
Target3	4	(121° W, 29° S)	visible light	0.5
Target4	3	(40° W, 11° S)	microwave	0.8
Target5	4	(113° E, 49° S)	microwave	0.7
Target6	5	(160° W, 55° S)	visible light	0.6
Target7	4	(32° E, 26° N)	microwave	0.8
Target8	2	(89° E, 33° S)	microwave	0.9
Target 9	3	(153° W, 27° S)	microwave	0.9
Target10	3	(112° E, 12° S)	microwave	0.9
Target11	5	(36° W, 19° S)	visible light	0.4
Target12	4	(9° W, 60° N)	microwave	0.8
Target13	2	(7° W, 47° N)	visible light	0.5
Target14	4	(119° W, 51° N)	microwave	0.8
Target15	2	(78° E, 43° N)	microwave	0.9
Target16	4	(89° W, 9° N)	microwave	0.8
Target17	4	(141° E, 2° S)	visible light	0.5
Target18	2	(42° W, 32° N)	visible light	0.4
Target19	1	(17° W, 49° N)	visible light	0.6
Target20	5	(78° E, 37° S)	microwave	0.7
Target21	3	(15° E, 18° N)	visible light	0.5
Target22	2	(43° E, 28° N)	microwave	0.7
Target23	2	(151° E, 4° N)	microwave	0.9
Target24	4	(137° W, 18° N)	microwave	0.8
Target25	4	(49° W, 44° N)	visible light	0.5
Target26	2	(13° W, 44° S)	microwave	0.9
Target27	1	(46° W, 45° N)	microwave	0.8
Target28	3	(113° E, 19° N)	visible light	0.3
Target29	2	(171° W, 44° S)	visible light	0.3
Target30	4	(35° W, 58° N)	visible light	0.4

**Table 5:** Emergency targets parameters.

Target ID	Priority	Geographical Position	Observation Type	Resolution Requirement
Target31	5	(102° W, 18° S)	visible light	0.4
Target32	5	(45° W, 17° N)	microwave	0.8
Target33	5	(7° E, 30° N)	microwave	0.7
Target34	5	(110° E, 32° S)	microwave	0.7
Target35	5	(86° W, 54° S)	visible light	0.6

**Table 6:** Satellite Information.

	S1	S2	S3	S4	S5	S6
Type	Sar	Opt	Sar	Sar	Opt	Opt
Resolution	0.5	0.3	0.7	0.7	0.5	0.3

**Table 7:** Initial scheduling plan.

Target ID	Priority	Satellite ID	Start Time	End Time
Target1	4	S4	0:33:27	0:37:36
Target2	4	S4	14:42:52	14:49:05
Target3	4	S5	22:48:53	22:51:50
Target4	3	S3	2:08:03	2:13:17
Target5	4	S3	17:52:24	17:58:03
Target6	5	S5	0:33:25	0:36:08
Target7	4	S1	10:22:50	10:29:23
Target8	2	S4	9:01:19	9:07:56
Target9	3	S4	12:48:30	12:54:09
Target10	3	S1	4:06:20	4:12:45
Target11	5	S6	17:23:42	17:26:11
Target12	4	S3	23:12:30	23:15:20
Target13	2	S5	16:01:22	16:04:26
Target14	4	S4	9:58:56	10:01:40
Target15	2	S1	17:45:33	17:51:46
Target16	4	S1	18:19:39	18:25:49
Target17	4	unscheduled	/	/
Target18	2	S2	14:42:15	14:44:01
Target19	1	S6	17:05:26	17:07:41
Target20	5	S3	19:32:21	19:38:55
Target21	3	S2	11:32:14	11:33:18
Target22	2	S4	11:59:12	12:05:39
Target23	2	S3	13:29:07	13:35:10
Target24	4	S1	9:48:14	9:49:59
Target25	4	S6	18:43:41	18:46:45
Target26	2	S1	12:18:22	12:20:52
Target27	1	S3	2:21:50	2:24:20
Target28	3	S6	7:44:52	7:47:51
Target29	2	S2	22:53:34	22:56:37
Target30	4	S2	14:34:52	14:37:03

**Table 8:** Dynamic scheduling plan.

Target ID	Priority	Satellite ID	Operation	Start Time	End Time
Target8	2	S4→S1	reschedule	17:27:06	17:29:43
Target12	4	S3→S1	reschedule	11:52:48	11:56:00
Target30	4	S2→none	delete	/	/
Target31	5	unscheduled	delete	/	/
Target32	5	S3	insert	15:48:54	15:52:55
Target33	5	S4	insert	15:11:49	15:18:07
Target34	5	S4	insert	7:28:31	7:30:58
Target35	5	S2	insert	16:29:58	16:32:00

**Table 9:** Simulation results of dynamic scheduling.

Case No.	Satellite Number	Initial Targets Number	Malfunction Targets	Newly Arrived Targets	*CR*	*PR*	*IR*	*ER*	Run Time of Initial Plan(s)	Run Time of Dynamic Plan(s)	Evaluation Function
No.1	3	25	0	5	0.967	0.969	0	1	4.2	1.2	0.98
No.2	6	50	5	10	0.950	0.974	0.102	0.867	5.3	1.5	0.92
No.3	8	50	10	20	0.971	0.981	0.22	0.967	6.1	2.4	0.92
No.4	8	100	15	30	0.915	0.932	0.196	0.911	18.7	3.7	0.89
No.5	12	100	15	35	0.933	0.938	0.188	0.96	20.2	5.3	0.91
No.6	16	200	20	40	0.979	0.981	0.12	0.983	31.4	8.1	0.96

## Data Availability

The data that support the findings of this study are available from the corresponding author upon reasonable request.

## Appendix A

The pseudo code for APSOA is shown as follows:

**Algorithm A1 APSOA**
**Input**: Targets Set *T_i_*, acceleration coefficients *c_1_*,*c_2_*, inertia weighted parameters, last action *a_i_^t−1^*, last payoff *U_i_^t−1^*, **Output**: *U_i_^t^*, *BR_i_^t^* **Procedure**:1. Randomly generate current swarm according to *T_i_*; 2. Insert last action *a_i_^t−1^* to current swarm; 3. **for** each iteration *g* = 1,2… **do** 4. **for** each particle *p* = 1,2… **do** 5. De-conflict and calculate the profit; 6. Compare and substitute the individual optimal and global optimal; 7. Update particle’s location and velocity in (16); 8. Boundary condition processing; 9. **end for** 10. **end for** 11. Compare the best *U_i_^t^* of swarm with *U_i_^t−1^*, and select *a* with higher *U_i_* as *BR_i_^t^*;

(A1) $$\begin{array}{c} v_{ij}(t+1)=\lambda \cdot v_{ij}(t)+c_{1}r_{1}(t)[p_{ij}(t)-x_{ij}(t)]+c_{2}r_{2}(t)[p_{gj}(t)-x_{ij}(t)]\\ x_{ij}(t+1)=x_{ij}(t)+v_{ij}(t+1) \end{array}$$

where particle population *P = (X_1_*, *X_2_*,*…*, *X_N_)* is randomly generated, and each particle *X_i_ = (x_i1_*, *x_i2_*,*…*, *x_iD_)* is a solution that will be further iterated. *D* is the dimension of the solution (it refers to the number of targets in this paper), and all the components in *X_i_* together determine the position of *X_i_*. Each particle has a velocity *V_i_ = (v_i1_*, *v_i2_*,*…*, *v_iD_)*; velocity controls the speed and direction of particle evolution. The individual optimal so far is *p_best_ = (p_i1_*, *p_i2_*,*…*, *p_iD_)*, and the global optimal is *g_best_ = (g_1_*,*g_2_*,*…*,*g_D_)*.

## Appendix B

The pseudo code for ATSA is shown as follows:

**Algorithm A2 ATSA**
**Input**: Targets Set *T_i_*, neighborhood size *Ca*, termination parameters, last action *a_i_^t−1^*,last payoff *U_i_^t−1^*, **Output**: *U_i_^t^*, *BR_i_^t^* **Procedure**: 1. **for** each satellite *s* = 1,2… **do** 2. Find allocated but unscheduled targets and update solution structure; 3. **for** each global iteration *g* = 1,2… **do** 4. Initialize local iteration parameters, including tabu list length, localmaximum iteration number, etc.; 5. **for** each local iteration *l* = 1,2… **do** 6. Generate the neighborhood solution and calculate payoff respectively, and retain the solution with maximum as candidate solution; 7. Update the current solution with candidate solution and best solutionso far with the candidate solution if aspiration judgement is satisfied; 8. Update tabu-list; 9. **end for** 10. **end for** 11. **end for** 12. Compare the best *U_i_^t^* with *U_i_^t−1^*, and select *a* with higher *U_i_* as *BR_i_^t^*;

## References

[1] Araguz C., Llaveria D., Lancheros E., Bou-Balust E., Camps A., Alarcon E., Lluch I., Matevosyan H., Golkar A., Tonetti S. (2018). Optimized Model-Based Design Space Exploration of Distributed Multi-Orbit Multi-Platform Earth Observation Spacecraft Architectures.

[2] Rajah P.M., Prokopenko M., Wang P., Price D. On Decentralised Clustering in Self-Monitoring Networks. Proceedings of the fourth International Joint Conference on Autonomous Agents & Multiagent Systems.

[3] Abbott D., Doyle B., Dunlop J., Farmer T., Hedley M., Herrmann J., James G., Johnson M., Joshi B., Poulton G. (2002). Development and Evaluation of Sensor Concepts for Ageless Aerospace Vehicles: Development of Concepts for an Intelligent Sensing System.

[4] Prokopenko M., Wang P., Price D. Towards Adaptive Clustering in Self-monitoring Multi-agent Networks. Proceedings of the International Conference on Knowledge-Based Intelligent Information & Engineering Systems.

[5] Araguz C., Bou-Balust E., Alarcon E. (2018). Applying autonomy to distributed satellite systems: Trends, challenges, and future prospects. Syst. Eng..

[6] Zhang S., Xiao Y., Yang P., Liu Y., Chang W., Zhou S. (2019). An Effectiveness Evaluation Model for Satellite Observation and Data-Downlink Scheduling Considering Weather Uncertainties. Remote Sens..

[7] Sun C., Wang X., Liu X. Distributed Satellite Mission Planning via Learning in Games. Proceedings of the 2018 IEEE International Conference on Systems, Man, and Cybernetics (SMC).

[8] Iacopino C., Palmer P., Policella N., Donati A., Brewer A. (2014). How ants can manage your satellites. Acta Futur..

[9] Kilic S., Ozkan O. Ant colony optimization approach for satellite broadcast scheduling problem. Proceedings of the 2017 8th International Conference on Recent Advances in Space Technologies (RAST).

[10] Tripp H., Palmer P. (2010). Stigmergy based behavioural coordination for satellite clusters. Acta Astronaut..

[11] Wu G., Liu J., Ma M., Qiu D. (2013). A two-phase scheduling method with the consideration of task clustering for earth observing satellites. Comput. Oper. Res..

[12] Bunkheila F., Circi C. (2018). Innovative Satellite Scheduling Method Based on Genetic Algorithms and Simulated Annealing and Related Mission Planner. EP Patent.

[13] Xhafa F., Sun J., Barolli A., Biberaj A., Barolli L. (2012). Genetic algorithms for satellite scheduling problems. Mob. Inf. Syst..

[14] Greco C., Gentile L., Filippi G., Minisci E., Vasile M., Bartz-Beielstein T. Autonomous generation of observation schedules for tracking satellites with structured-chromosome GA optimisation. Proceedings of the 2019 IEEE Congress on Evolutionary Computation (CEC).

[15] Mansour M.A., Dessouky M.M. (2010). A genetic algorithm approach for solving the daily photograph selection problem of the SPOT5 satellite. Comput. Ind. Eng..

[16] Cui J., Zhang X. (2019). Application of a multi-satellite dynamic mission scheduling model based on mission priority in emergency response. Sensors.

[17] Bianchessi N., Cordeau J.-F., Desrosiers J., Laporte G., Raymond V. (2007). A heuristic for the multi-satellite, multi-orbit and multi-user management of earth observation satellites. Eur. J. Oper. Res..

[18] Vasquez M., Hao J.-K. (2001). A “logic-constrained” knapsack formulation and a tabu algorithm for the daily photograph scheduling of an earth observation satellite. Comput. Optim. Appl..

[19] Ocon J. Multi-agent frameworks for space applications. Proceedings of the SpaceOps 2010 Conference Delivering on the Dream Hosted by NASA Marshall Space Flight Center and Organized by AIAA.

[20] Wang C., Li J., Jing N., Wang J., Chen H. (2011). A Distributed Cooperative Dynamic Task Planning Algorithm for Multiple Satellites Based on Multi-agent Hybrid Learning-ScienceDirect. Chin. J. Aeronaut..

[21] Bonnet J., Gleizes M.P., Kaddoum E., Rainjonneau S., Flandin G. Multi-satellite mission planning using a self-adaptive multi-agent system. Proceedings of the 2015 IEEE 9th International Conference on Self-Adaptive and Self-Organizing Systems.

[22] Hess J.A., Saunders D., Cobb R.G., Zagaris C. Autonomous Cooperative Optimal Control of Multi-Agent Satellite Formations. Proceedings of the 2020 AAS/AIAA Astrodynamics Specialist Virtual Lake Tahoe Conference.

[23] Araguz C., Closa M., Bou-Balust E., Alarcon E. A Design-Oriented Characterization Framework for Decentralized, Distributed, Autonomous Systems: The Nano-Satellite Swarm Case. Proceedings of the 2019 IEEE International Symposium on Circuits and Systems (ISCAS).

[24] Kuhn H.W. (1997). Classics in Game Theory.

[25] Başar T., Baillieul J., Samad T. (2013). Game Theory: Historical Overview. Encyclopedia of Systems and Control.

[26] Nash J.F. (1951). Non-Cooperative Games; Annals of Mathemtics.

[27] Kusyk J., Uyar M.U., Ma K., Samoylov E., Boksiner J. (2020). Artificial intelligence and game theory controlled autonomous UAV swarms. Evol. Intell..

[28] Xing G., Chen Y., Hou R., Dong M., Ma M. (2021). Game Theory-based Clustering Scheme for Energy Balancing in Underwater Acoustic Sensor Networks. IEEE Internet Things J..

[29] Cintuglu M.H., Martin H., Mohammed O.A. (2017). Real-Time Implementation of Multiagent-Based Game Theory Reverse Auction Model for Microgrid Market Operation. IEEE Trans. Smart Grid.

[30] Kasthurirathna D., Piraveenan M. (2015). Emergence of scale-free characteristics in socio-ecological systems with bounded rationality. Sci. Rep..

[31] Kasthurirathna D., Piraveenan M., Uddin S. (2016). Modeling networked systems using the topologically distributed bounded rationality framework. Complexity.

[32] Piraveenan M. (2019). Applications of Game Theory in Project Management: A Structured Review and Analysis. Mathematics.

[33] Pemberton J.C., Greenwald L. (2002). On the need for dynamic scheduling of imaging satellites. Int. Arch. Photogramm. Remote Sens. Spat. Inf. Sci..

[34] Bensana E., Lemaitre M., Verfaillie G. (1999). Earth observation satellite management. Constraints.

[35] Gabrel V., Vanderpooten D. (2002). Enumeration and interactive selection of efficient paths in a multiple criteria graph for scheduling an earth observing satellite. Eur. J. Oper. Res..

[36] Dishan Q., Chuan H., Jin L., Manhao M. (2013). A dynamic scheduling method of earth-observing satellites by employing rolling horizon strategy. Sci. World J..

[37] He L., Liu X.-L., Chen Y.-W., Xing L.-N., Liu K. (2019). Hierarchical scheduling for real-time agile satellite task scheduling in a dynamic environment. Adv. Space Res..

[38] Wang J., Zhu X., Yang L.T., Zhu J., Ma M. (2015). Towards dynamic real-time scheduling for multiple earth observation satellites. J. Comput. Syst. Sci..

[39] Jiang A.X., Leyton-Brown K. (2009). A Tutorial on the Proof of the Existence of Nash Equilibria.

[40] Song Y.J., Wang P., Zhang Z.S., Xing L.N., Chen Y.W. (2019). An Improved Genetic Algorithm for Multi-Satellite Mission Planning Problem.

[41] Han Y., Luo J., Xu X. (2019). On the Constellation Design of Multi-GNSS Reflectometry Mission Using the Particle Swarm Optimization Algorithm. Atmosphere.

